# Characteristics of strokes associated with centrifugal flow left ventricular assist devices

**DOI:** 10.1038/s41598-021-81445-8

**Published:** 2021-01-18

**Authors:** Ovais Inamullah, Yuting P. Chiang, Muath Bishawi, Martin Weiss, Michael W. Lutz, Laura J. Blue, Wayne Feng, Carmelo A. Milano, Matthew Luedke, Nada El Husseini

**Affiliations:** 1 Department of Neurology, Duke University Medical Center, 2301 Erwin Road, Durham, NC 27710 USA; 2 Division of Cardiothoracic Surgery, Department of Surgery, Duke University Medical Center, Durham, USA

**Keywords:** Neuroscience, Diseases of the nervous system, Neurology, Neurological disorders, Cardiac device therapy, Cardiovascular biology

## Abstract

Stroke is a devastating complication of left ventricular assist device (LVAD) therapy. Understanding the characteristics, risk factors and outcomes of strokes associated with the centrifugal flow LVADs is important to devise better strategies for management and prevention. This is a retrospective cohort study at a single US academic medical center. The cohort includes patients who received a first time Heartmate 3 (HM3) or Heartware (HVAD) LVAD between September 2009 through February 2018 and had a stroke while the LVAD was in place. Descriptive statistics were used when appropriate. A logistic regression analysis was used to determine predictors of poor outcome. Out of a total of 247 patients, 12.1% (N = 30, 24 HVAD and 6 HM3) had a stroke (63% ischemic) and 3 of these patients had pump thrombosis. Events per patient year (EPPY) were similar for HVAD and HM3 patients (0.3 ± 0.1). INR was subtherapeutic in 47.4% of ischemic stroke patients and supratherapeutic in 18.2% of hemorrhagic stroke patients. Concurrent infections were more common in the setting of hemorrhagic stroke than ischemic stroke (45.4% vs 5.3%, p = 0.008). Strokes were severe in most cases, with initial NIH stroke scale (NIHSS) higher in HM3 patients compared to HVAD patients (mean 24.6 vs 16) and associated with high in-patient mortality (21.1% of ischemic stroke vs. 88.8% of hemorrhagic stroke). Predictors of death within 30 days and disability at 90 days included creatinine at stroke onset, concurrent infection, hemorrhaghic stroke, and initial stroke severity (NIHSS). A score derived from these variables predicted with 100% certainty mortality at 30 days and mRS ≥ 4 at 90 days. For patients with centrifugal flow LVADs, ischemic strokes were more common but hemorrhagic strokes were associated with higher in-patient mortality and more frequently seen in the setting of concurrent infections. Infections, sub or supratherapeutic INR range, and comorbid cardiovascular risk factors may all be contributing to the stroke burden. These findings may inform future strategies for stroke prevention in this population.

## Introduction

Implantation of a mechanical pump to assist with cardiac output, also known as a left ventricular assist device (LVAD), is often used in the management of medically refractory advanced heart failure. An LVAD can be placed while the patient is awaiting a heart transplant, known as bridge to transplant (BTT), or can be used as a permanent therapy for advanced heart failure patients that are poor transplant candidates, known as destination therapy (DT). Previous studies showed that LVAD can improve long term survival and quality of life for these patients^1–3^.

Stroke, however, is a devastating complication of LVAD therapy and a leading cause of mortality and morbidity in this group^4,5^. A stroke can significantly reduce the odds of an LVAD patient receiving a heart transplant and is associated with a shorter life expectancy and poor quality of life^6^. Therefore, stroke prevention is a crucial part of the medical management of LVAD patients. The rate of stroke in LVAD patients has previously been reported to be between 10 and 20%, but this frequency varies depending on the type of LVAD^7^. Stroke in this population can be attributed to clot formation in the LVAD, debris in the outflow graft, carotid or intracranial artery disease, or cardioembolic from the left atrium, left ventricle, or aortic arch. Stroke risk in this population is likely elevated due to higher comorbid cardiovascular risk factors and other physiologic changes that occur in the setting of significant heart failure.

The HeartWare (HVAD, 2012) and HeartMate 3 (HM3, 2017) devices use a continuous flow centrifugal pump with a magnetically levitated rotor that may cause less pump thrombosis. Based on the MOMENTUM 3 trial, the HM3 may have a lower stroke risk compared to prior axial flow devices like the HM2^8^. Patients who received the centrifugal flow HM3 device had lower stroke risks compared to the HM2 device (10% vs 19%) but had similar risks of death and disabling stroke^8^. On the other hand, data from the ADVANCE and ENDURANCE trials showed an elevated risk of stroke for the HVAD device compared to the HM2^9,10^. The trials, however, are difficult to compare due to differences in their design including patient selection criteria and definitions of outcomes.

Previous studies of stroke in the setting of LVAD have mostly included older axial flow LVADs. First generation LVADs like the HeartMate XVE have been shown to have higher stroke rates at around 25%, especially in patients with concurrent infection or prolonged use of an LVAD^11^. A study of HeartMate 2 (HM2; Abbott Inc., Chicago IL) patients showed a stroke frequency of 12%, with two thirds of these strokes being ischemic^12^. Another study showed a stroke frequency of 17% in patients with HeatMate2, with twice the risk of mortality compared to those without stroke^13^. Ischemic strokes occur more often in LVAD patients then hemorrhagic strokes, but both types of strokes have significant mortality risks^14^. The high mortality seen in LVAD patients who have strokes makes it imperative to better understand the stroke risks in this unique population and to devise strategies for optimal stroke prevention and management^15^.

The stroke risk in centrifugal flow LVADs has been previously described^16^. In this study, out of a total of 247 patients with HVAD or HM3 device, 30 strokes occurred with, a higher frequency in HVAD patients (14.7%) compared to HM3 patients (7.1%). Using the same cohort, we sought to compare the stroke risk between the devices, to identify modifiable risk factors that could inform future stroke prevention strategies, and to evaluate for predictors of post-stroke mortality and functional outcomes.

## Methods

This was a retrospective cohort study at a single US academic medical center. This study was approved by the Institutional Review Board at Duke University. The cohort includes all subjects over the age of 18 years at Duke University Medical Center who received a first time HM3 or HVAD LVAD between September 2009 through February 2018 and had a cerebrovascular accident while the LVAD was in place. Patients with pre-operative ECMO were excluded from the analysis as this could be a confounder for stroke etiology. Patients were followed using chart review until December 31^st^, 2019. All methods were carried out in accordance with relevant guidelines and regulations.

Each LVAD was placed by a cardiothoracic surgeon at Duke University. All surgeries were done via a median sternotomy, with the outflow tract anastomosed to the ascending aorta. The inflow tract is connected to the apex of the left ventricle. Diagnosis of stroke was based on clinical and imaging data. Ischemic and hemorrhagic strokes were included. Only intracerebral hemorrhage was included; isolated subdural hematoma or subarachnoid hemorrhage was not included. Patients were initially anticoagulated postoperatively with heparin with a goal PTT of 60–90, and then patients were transitioned to a regimen of aspirin 325 mg daily and warfarin for a target INR of 2–3. Any INR < 2 was considered subtherapeutic and any INR > 3 was considered supratherapeutic. No other antiplatelet drugs were used. Diagnosis of pump thrombosis was based on evidence of a thrombus on echocardiogram, a device exchange for suspected thrombosis, or made clinically based on patient symptoms, evidence of hemolysis, and changes in pump power usage. Stroke etiology was based on neurology documentation in the medical records and classified according to the TOAST criteria^17^.

Data regarding patient demographics, risk factors and comorbidities, stroke location and characteristics, vascular imaging results, LVAD surgical information, and management decisions were obtained from the retrospective review of the electronic medical system. No prospective data was used. As LVAD patients are unable to get MRI’s, imaging information was obtained from CT scans. Labs and vitals at stroke onset refer to the first values recorded after onset of acute neurological symptoms. Degree of stenosis on vascular imaging was determined by neuroradiologist report. Follow-up time was calculated from date of surgery until death or last date of chart review using the LVAD database on 12/31/2019. Events per patient year (EPPY) was calculated using total number of ischemic or hemorrhagic strokes per patient, divided by number of years from LVAD implantation until final chart review on 12/31/2019. This information was obtained from chart review; patients were not prospectively followed. No patient consent was obtained since this was a retrospective study; this was approved by the Institutional Review Board and the ethics committee at Duke University.

Descriptive statistics were calculated to compare the clinical variables for individuals receiving either the HVAD or HM3 device. A t-test was used for continuous variables and Chi- square test for categorical variables. P-values were calculated to compare variables in HVAD and HM3 patients. Statistical analysis was done on JMP Pro 14. A p value of ≤ 0.05 was considered statistically significant.

**Figure 1:**
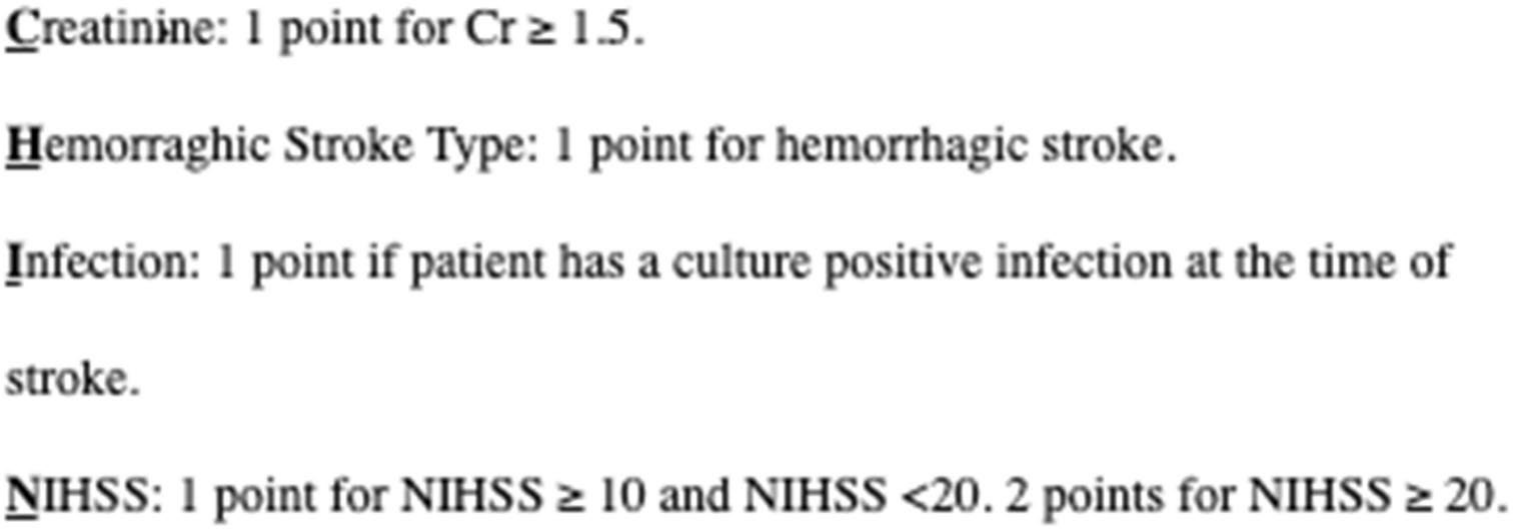
CHIN Risk Score from 0–5 is used to predict likelihood of poor outcome after stroke in centrifugal flow LVAD patients with initial NIHSS ≥ 5. The elements of the CHIN risk score are given.

A logistic regression analysis was then used to determine predictors of poor outcome. Poor outcome was defined as a modified Rankin Scale (mRS) of 4 or worse at 90 days or death within 30 days. Patients with minor strokes at onset (initial NIHSS < 5) were excluded from this analysis to focus on patients at high risk of disability and mortality. The variables that were determined to correlate with poor outcome were combined to form the CHIN (Creatinine, Hemorrhage, Infection, NIHSS) risk score shown in Fig. 1. A contingency analysis with a Chi-square test was used to show the CHIN risk score correlation with poor outcomes in this patient cohort.

## Results

### Demographics and risk factors

**Figure 2:**
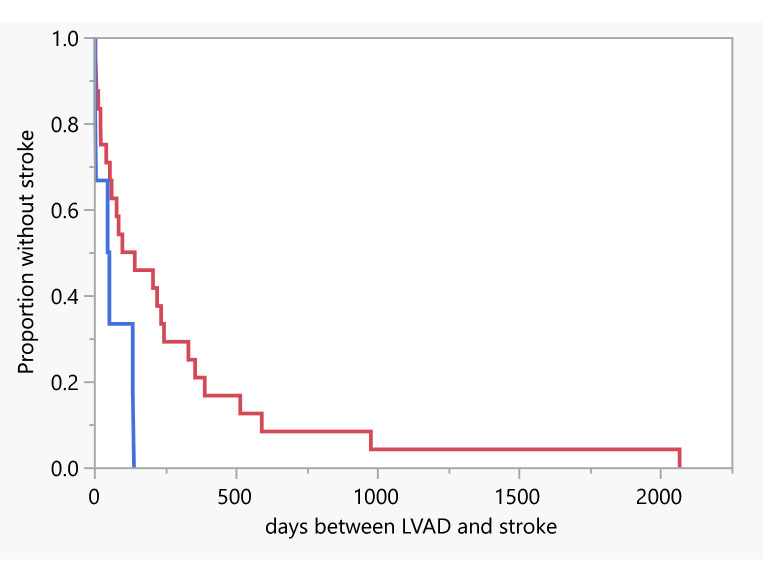
Kaplan Meier analysis of days from LVAD implantation to first stroke for each device (Red = HVAD, Blue = HM3). Using the log rank test, this difference was significant with p = 0.0297.

Out of a total of 247 patients (163 HVADs and 84 HM3s), 30 (12.1%) were diagnosed with an acute stroke. Demographics and characteristics of these patients are outlined in Table 1. Of the 30 total strokes, 19 (63.3%) were ischemic and 11 (36.7%) were hemorrhagic. Average follow-up time in days was slightly longer in HVAD patients compared to HM3 patients (645.9 vs 505.3), but this difference was not significant. Events per patient year (EPPY) were similar for HVAD and HM3 patients (0.3 ± 0.1). A Kaplan Meier analysis for time from LVAD implantation to first stroke for each device is shown in Fig. 2 and shows the HM3 population with earlier strokes. Using the log rank test, this difference was significant with p = 0.030, but the comparison is limited by the small sample size.

**Table 1:** Patients’ clinical and demographical characteristics.

Variables	HVAD	HM3	p-value
Number of patients	163	84	–
Number of strokes	24 (14.7%)	6 (7.1%)	0.0836
Ischemic strokes [no (%)]	15 (62.5%)	4 (66.7%)	0.8493
Follow up time in days [mean ± SD]	645.9 ± 783.3	505.3 ± 691.1	0.675
EPPY	0.3 ± 0.1	0.3 ± 0.1	0.8584
Age in years [mean ± SD]	61.4 ± 12.5	73.5 ± 4.3	0.001
Female [no (%)]	4 (16.7%)	2 (33.3%)	0.269
**Race [no (%)]**			
White	19 (79.2%)	4 (66.7%)	0.644
Black	4 (16.7%)	2 (33.3%)	
Other	1 (4.2%)	0 (0%)	
Destination LVAD^a^ [no (%)]	18 (75.0%)	5 (83.3%)	0.283
**Co-morbidities [no (%)]**			
Ischemic heart failure	12 (50.0%)	2 (33.3%)	1.000
Prior CVA or TIA	4 (16.7%)	0 (0.0%)	1.000
Prior PE or DVT	2 (8.3%)	1 (16.7%)	0.446
Diabetes	9 (37.5%)	3 (50.0%)	0.624
Hypertension	14 (58.3%)	4 (66.7%)	1.000
Hyperlipidemia	14 (58.3%)	4 (66.7%)	1.000
Atrial fibrillation	11 (45.8%)	4 (66.7%)	0.632
COPD	5 (20.8%)	1 (16.7%)	1.000
Previous or current tobacco user	11 (45.8%)	3 (50.0%)	0.651
**Labs/vitals**^**b**^			
MAP (mean ± SD)	82.7 ± 17.6	113.6 ± 25.5	0.320
PTT (mean ± SD)	43.4 ± 17.2	32.9 ± 3.5	0.468
INR (mean ± SD)	2.3 ± 1.2	1.9 ± 0.8	0.520
INR 2.0–3.0 [No (%)]	8 (33.3%)	2 (33.3%)	1.000
Creatinine (mean ± SD)	1.5 ± 0.9	1.3 ± 0.5	0.367
**LVAD complications at the time of stroke**^**c**^** [no (%)]**			
GI Bleed	1 (4.2%)	0 (0.0%)	0.675
Pump thrombosis	3 (12.5%)	0 (0.0%)	1.000
Infection^d^	5 (20.8%)	1 (16.7%)	1.000

^a^This refers to the LVAD being placed as destination therapy. All other patients had LVAD placed as bridge to transplant.

^b^First set of labs and vitals after onset of neurological symptoms were used.

^c^“At the time of stroke” refers to the onset of neurological symptoms corresponding to the diagnosed stroke.

^d^Infection included any culture positive bacterial infection at the time of stroke diagnosis.

Patients with HM3 were older and had a higher rate of most cardiovascular co-morbidities including hypertension, diabetes, atrial fibrillation, and smoking history. All of the patients with HM3 who had a stroke had destination LVADs, whereas in the HVAD group, strokes occurred in those with destination therapy or bridge to transplant. Only 10% of the patient cohort had pump thrombosis. None of the patients with pump thrombosis were candidates for thrombolysis. Pump thrombosis was identified in 12.5% of HVAD stroke patients and a preceding GI bleed that required holding anticoagulation was seen in 4.2% of HVAD stroke patients. No patients in the HM3 group had pump thrombosis or GI bleeding at the time on stroke onset.

The most common medical co-morbidities identified were hypertension, diabetes, hyperlipidemia, and a history of smoking. Half of the patients had atrial fibrillation thus increasing the risk for cardioembolic strokes. The HM3 stroke patients were more likely to be females and not white, were older, and had more diabetes and atrial fibrillation. Initial mean arterial pressure (MAP) appeared to trend higher in HM3 stroke patients (mean 113.6 vs 82.7, p = 0.0320). Creatinine during stroke presentation was similar between HM3 and HVAD patients (mean 1.5 vs 1.3, p = 0.367).

Patients without a therapeutic INR or PTT at the time of stroke presentation comprised 60% of this patient cohort. This was for a variety of reasons including patient noncompliance, infections, or warfarin being held for bleeding or a procedure. In ischemic stroke patients, INR was subtherapeutic for 47.4% of patients and supratherapeutic for 21.1% of patients. In hemorrhagic stroke patients, INR was subtherapeutic for 27.3% of patients and supratherapeutic in 18.2% of patients. All four HM3 ischemic stroke patients had subtherapeutic INRs, while both of the HM3 hemorrhagic stroke patients had an INR in the therapeutic range.

## Infections

A culture positive bacterial infection was present in 6 (20%) of the 30 stroke patients. These infections met criteria for a major infection for VAD patients by the INTERMACS registry group^18^. Of these six infections that led to stroke in LVAD patients, only one was an ischemic stroke. The was a much higher percentage of infections seen in hemorrhagic stroke patients compared to ischemic stroke patients (45.4% vs 5.3%, p = 0.008).

Of the 6 patients with infection at the time of stroke, 4 patients had bacteremia, 1 had pneumonia, and 1 had a wound infection. The 1 patient with ischemic stroke that had an infection had MSSA bacteremia. There were 3 patients with bacteremia and hemorrhagic stroke; two patients had blood cultures positive for MRSA and the other patient had blood cultures positive for Strep Infantarius. The patient with hemorrhagic stroke and pneumonia had a bronchoalveolar lavage that showed mixed GNRs. Another patient with hemorrhagic stroke and a wound infection had a chest washout and culture that was positive for multidrug-resistant Achromobacter Xylosoxidans, and this patient was also diagnosed with a clostridium difficile gastrointestinal infection.

Each of these 6 patients was on antibiotics at the time of their stroke. All 6 were being treated with vancomycin, but the rest of the antibiotics varied. Piperacillin/Tazobactam was the second most common antibiotic and used in 3 patients. Other antibiotics used included cefepime, metronidazole, meropenem, ceftriaxone, ceftaroline, and Ertapenem. INR for the patients ranged from 1.5 to 2.3, and the average was 1.8. None of the patients had a supratherapeutic INR. Two of the patients with hemorrhagic stroke were on heparin (warfarin was held) at the time of the stroke with PTT’s of 59.6 and 40.9. Four of the six patients who had infections were also on aspirin at the time of stroke. Platelets for these 6 patients varied from 72 to 238, with an average of 187.

### Stroke characteristics and patient outcomes

Stroke characteristics including cerebral localization, vascular imaging results, suspected etiology, management decisions, and patient outcomes are shown in Table 2. Lab values and stroke severity and outcome assessments were obtained at the time of diagnosis of cerebrovascular accident.

**Table 2:** Stroke characteristics and patient outcomes.

Variable	HVAD	HM3	p-value
**Stroke location on imaging [no (%)]**			
Cortical	12 (50.0%)	2 (33.3%)	0.115
Subcortical supratentorial	5 (20.8%)	0 (0.0%)	
Infratentorial	4 (16.7%)	0 (0.0%)	
Multifocal	3 (12.5%)	4 (66.7%)	
**Stroke etiology**^**a**^** [no (%)]**			
Cardioembolic	10 (66.7%)	3 (75.0%)	0.922
Atherosclerosis	1 (6.7%)	0 (0.0%)	
Small vessel	1 (6.7%)	0 (0.0%)	
Undetermined	3 (20.0%)	1 (25.0%)	
**Vascular imaging [no (%)]**			
Mod-severe extracranial atherosclerosis	6 (25.0%)	1 (16.7%)	0.720
Mod-severe intracranial atherosclerosis	3 (12.5%)	0 (0.0%)	0.509
Large vessel occlusion	3 (12.5%)	0 (0.0%)	0.720
**Stroke timing**			
Inpatient^b^ [no (%)]	10 (41.7%)	4 (66.7%)	0.453
< 30 days post LVAD surgery [no (%)]	7 (29.2%)	2 (33.3%)	1.000
Number of days between LVAD surgery and stroke (mean ± SD)	281.4 ± 447.0	76.8 ± 60.0	0.041
**Stroke severity scores**			
NIHSS (mean ± SD)	16 ± 12.2	24.6 ± 10.3	0.149
ICH Score^c^ (mean ± SD)	2.8 ± 1.2	3 ± 1.4	0.427
**Stroke management**			
Thrombectomy	2 (8.3%)	0 (0.0%)	0.674
Hemicraniectomy	0 (0.0%)	0 (0.0%)	n/a
Hyperosmolar therapy	0 (0.0%)	3 (50%)	0.172
Comfort care	10 (41.7%)	1 (16.7%)	0.632
**Patient outcomes**			
mRS ≥ 4 at 90 days	12 (50.0%)	5 (83.3%)	0.142
Death within 30 days	6 (25%)	1 (16.7%)	0.667

^a^Only ischemic strokes are included for these variables.

^b^Inpatient refers to any stroke occurred while patient was hospitalized either post-opertively from LVAD surgery or for a separate issue prior to onset of stroke.

^c^Only hemorrhagic strokes are included for this variable.

For ischemic strokes, etiology was most likely to be identified as cardioembolic or undetermined based on TOAST classification. Strokes in HVAD patients were most commonly cortical (50.0%), whereas strokes in HM3 patients were most commonly multifocal (66.7%). HVAD patients had a higher percentage of moderate or severe large vessel atherosclerosis, and more large vessel occlusions were seen. Vascular imaging was done in 70.8% of HVAD patients and 50.0% of HM3 patients. Moderate or severe vascular stenosis (intracranial or extracranial) was found in 33.3% of HVAD patients and 16.7% of HM3 patients (p = 0.423). An anterior circulation large vessel occlusion was found in 20% of the HVAD ischemic stroke patients compared to 0% in the HM3 group (p = 0.332).

Of the three HVAD ischemic stroke patients with ischemic stroke and HVAD that were found to have an acute large vessel occlusion, two received mechanical thrombectomy. Of the two patients that received thrombectomy, one had a good outcome with a discharge NIHSS of 4, and the other had a severe stroke and died prior to discharge. Hemicraniectomy was not done on any patients and medication to treat elevated intracranial pressure was used in 1 HM3 patient.

The initial NIH stroke scale (NIHSS) trended higher in HM3 patients compared to HVAD patients (mean 24.6 vs 16, p = 0.149). For patients with hemorrhagic stroke, ICH score was very similar between the HM3 and HVAD cohorts (mean 3 vs 2.8, p = 0.427). Inpatient strokes comprised 46.7% of this patient cohort. Inpatient stroke refers to a patient that had onset of symptoms and stroke diagnosed while the patient was already hospitalized for a separate issue. This includes patients who had a stroke while they were still in the hospital after their LVAD surgery.

The number of days between LVAD surgery and stroke was shorter in HM3 patients (mean 76.8 vs 281.4, p = 0.041). This is despite a similar proportion of strokes in the first 30 days after the LVAD surgery (33.3% vs 29.2%, p = 1.000). A decision was made in the acute setting to pursue comfort care with the patient expiring in 50.0% of HM3 patients and 41.7% of HVAD patients (p = 0.632). This decision was made in more hemorrhagic stroke patients compared to ischemic stroke patients (66.7% vs 37.5%, p = 0.001).

### Stroke distribution

**Figure 3:**
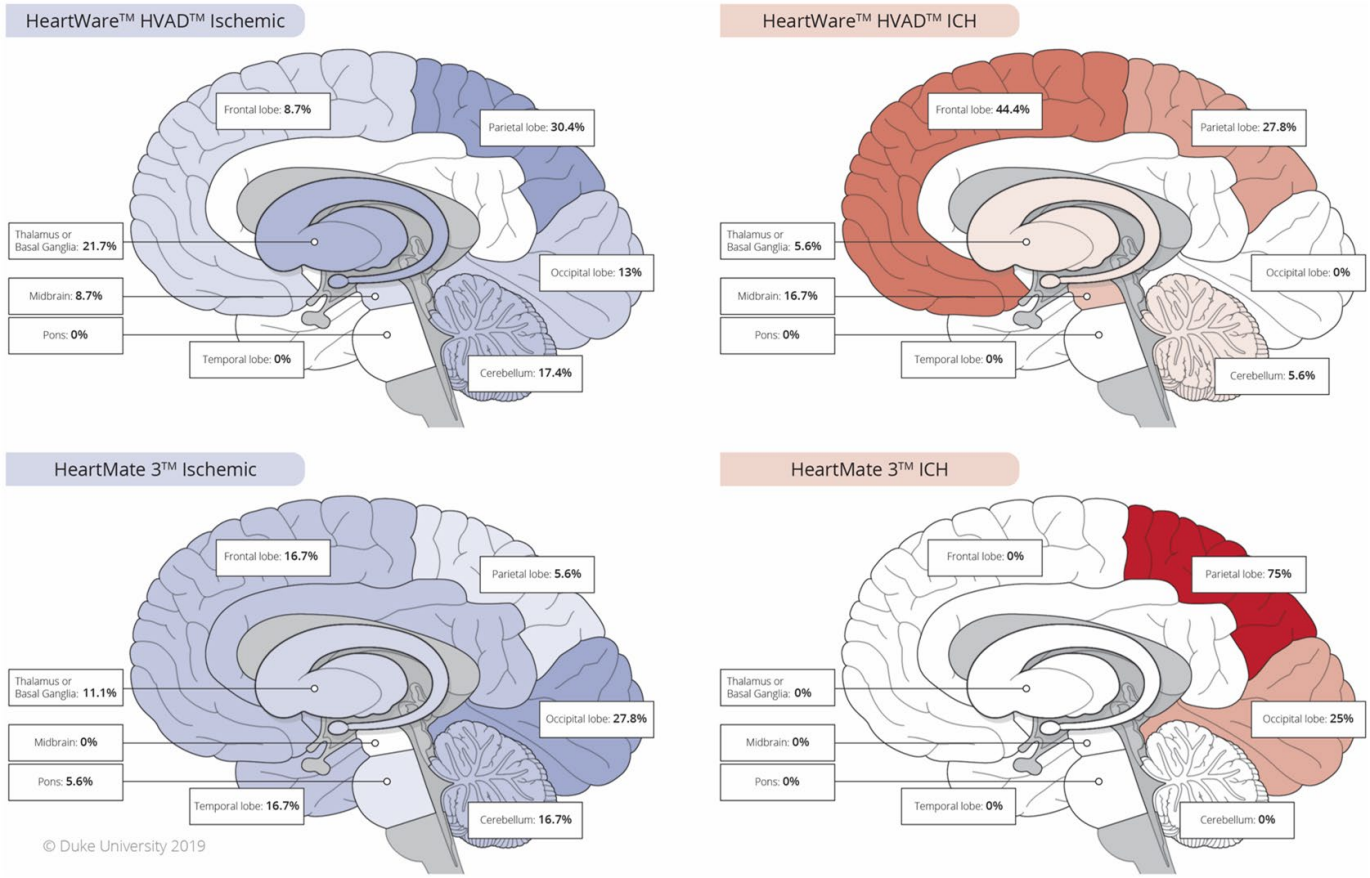
This graphic shows where strokes were identified for LVAD patients on cerebral imaging. If there were strokes in multiple locations for a patient, each location was recorded separately. Ischemic strokes are shown in blue and hemorrhagic strokes are shown in red, and are shaded according to how frequently each area was affected. The differences in distribution were not statistically significant. This figure was produced by medical illustrators in the Duke University Cardiothoracic Surgery Department.

The specific distribution of strokes for HVAD and HM3 patients is depicted in Fig. 3. None of the differences in location were statistically significant.

### Predictors of poor outcome

In this cohort of centrifugal flow LVAD patients who had an ischemic or hemorrhagic stroke, 17 out of 30 patients (56.7%) had a mRS of 4 or worse at 90 days, and 7 out of 30 patients (23.3%) died within 30 days. After excluding 9 patients with minor strokes (NIHSS < 5 at onset), 15 of 21 patients (71.4%) had a mRS of 4 or worse at 90 days, and 5 out of 21 patients (23.8%) died within 30 days. There was no difference in these outcomes between the two devices.

**Figure 4:**
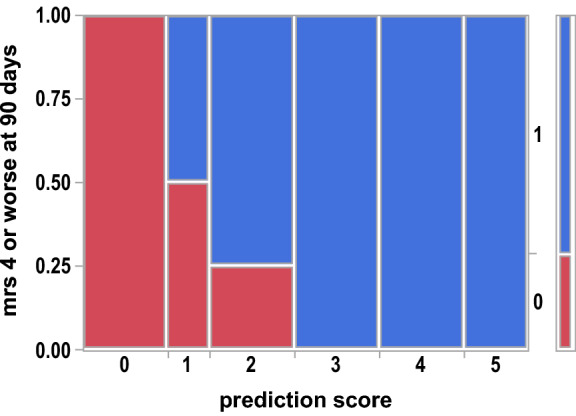
Contingency Analysis of mRS 4 or worse at 90 days by CHIN prediction score Mosaic Plot, p = 0.0031. Area represented by blue (1) is the proportion of patients that had an mRS of 4 or worse at 90 days.

**Figure 5:**
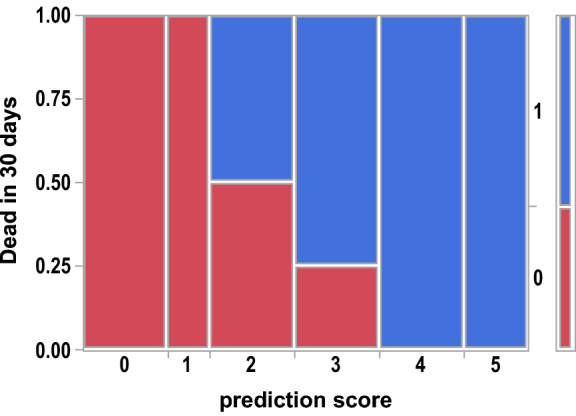
Contingency Analysis of Dead in 30 days by CHIN prediction score Mosaic Plot, p = 0.0022. Area represented by blue (1) is the proportion of patients that were dead within 30 days.

Risk factors for a poor outcome included creatinine at stroke onset, stroke type, concurrent infection, and initial stroke severity as measured by the NIHSS. The CHIN Risk score is shown in Figs. 4 and 5 to correlate with mRS of 4 or worse at 90 days and death within 30 days. A CHIN score of ≥ 3 was 100% associated with mRS of 4 or worse at 90 days and a CHIN score of ≥ 4 was 100% associated with death within 30 days.

## Discussion

We aimed to better understand stroke characteristics in the setting of the centrifugal flow LVADs (HM3 and HVAD devices). In this cohort, stroke risk in HM3 patients (7.2%) and HVAD patients (14.6%) appeared consistent with prior studies. HM3 patients were shown to have a 10.1% stroke risk in the MOMENTUM trial^8^, and HVAD patients were shown to have a 12.1% stroke risk in the ENDURANCE trial^10^. These are all lower then then 19.2% stroke risk demonstrated for the HeartMate II mechanical-bearing axial continuous flow pump in the MOMENTUM trial^8^. However, although stroke risk appeared lower in HM3 patients compared to HVAD patients in the initial analysis^16^, EPPY was similar between the two devices suggesting the difference may be due to longer follow-up times in the HVAD patients.

There was about a 2:1 ratio of ischemic strokes to hemorrhagic strokes, which was similar for both types of devices. With only 10% of the patient cohort having pump thrombosis, we suspect other vascular risk factors and stroke etiologies contributed to the strokes seen. HM3 stroke patients presented with a significantly higher MAP than HVAD patients. Data from the HVAD ADVANCE and ENDURANCE trials suggested that increased blood pressure was a significant predictor of stroke^9,10^. The HVAD ENDURANCE supplemental trial then demonstrated that enhanced control of BP in HVAD patients reduced stroke by 24.2%^19^. This suggests that more aggressive BP control may be beneficial, particularly for HM3 patients who had higher MAPs during stroke onset.

We carefully evaluated infections as well as antibiotic choice given possible interactions with warfarin to determine if infection was a significant risk factor for stroke in centrifugal flow LVAD patients. Infections were most commonly seen in hemorrhagic stroke patients. There were no patients with infections that had a supratherapeutic INR or PTT, so it was postulated that infections increased the risk of hemorrhagic stroke via an alternate mechanism. These patients were all on vancomycin which has previously been shown to have adverse effects on platelets^20^. Other explanations for bleeding during sepsis have been postulated including poor platelet function, deficiencies in coagulation factors, idiosyncratic drug reactions, bacterial endotoxins, vascular damage, and a “stress response”^21^.

INR or PTT was not therapeutic at the time of stroke presentation for more than half of this patient cohort. A nontherapeutic INR was seen for many reasons including anticoagulation being held for a procedure, anticoagulation being held for active bleeding, and INR changes in the setting of infection. This suggests that a large number of these strokes, including many of the devastating hemorrhagic strokes, may be preventable with infection control and closer monitoring of anticoagulation.

Comfort care was pursued in nearly half of the patients in this cohort. These strokes have clinical as well as financial implications. Quality of life can be reduced significantly as the patient acquires disability from a stroke. Patients frequently have prolonged hospitalizations after stroke which is associated with significant healthcare costs. Since stroke drastically changes the goals of care for this vulnerable population, it highlights the importance of primary stroke prevention in this group. Stroke risk stratification should be part of management plan before LVAD implantation.

For centrifugal flow LVAD patients who had an ischemic or hemorrhagic stroke with initial NIHSS ≥ 5, the CHIN score helped predict the risk of disability at 90 days or death within 30 days. For LVAD patients, hemorrhagic stroke compared to ischemic stroke has been previously associated with worse outcomes^22^, but the other variables including creatinine at stroke onset and concurrent infection are relatively novel predictors of outcomes in this population. Further prospective validation of this risk score is needed to confirm the findings and apply the risk score in the clinical setting.

There were several limitations in this study including a small sample size, a large age difference between HVAD and HM3 patients’ groups, and the retrospective nature of the study. There were only 6 patients in the HM3 patient cohort which limits comparisons. The small sample size and these factors may limit the generalizability of these findings. In addition, differences between device groups could potentially be explained by age or mere chance.

## Conclusion

This study provides valuable information about risk factors and stroke characteristics in patients with the centrifugal flow LVADs. We have identified multiple modifiable factors that affect stroke risk including infections, elevated blood pressure, acute illness leading to hospitalization, device thrombosis, and non-therapeutic anti-coagulation. These findings set the stage for future prospective clinical trials to decrease stroke occurrence in this population.

## References

[1] Pagani FD, Miller LW, Russell SD, Aaronson KD, John R, Boyle AJ, Conte JV, Bogaev RC, MacGillivray TE, Naka Y, Mancini D, Massey T, Chen L, Klodell CT, Aranda JM, Moazami N, Ewald G, Farrar DJ, Frazier OH (2009). Extended mechanical circulatory support with a continuous-flow rotary left ventricular assist device. J. Am. Coll. Cardiol..

[2] Miller, L. W., Pagani, F. D., Russell, S. D., John, R., Boyle, A. J., Aaronson, K. D., Conte, J.V., Naka, Y., Mancini, D., Delgado. R.M., MacGillivray, T. E., & Farrar, D.J. Use of a continuous-flow device in patients awaiting heart transplantation. *N. Engl. J. Med.* **357**(9), 885–896 (2007). 10.1056/NEJMoa067758 17761592

[3] Slaughter MS, Rogers JG, Milano CA, Russell SD, Conte JV, Feldman D, Sun B, Tatooles AJ, Delgado RM, Long JW, Wozniak TC, Ghumman W (2009). Advanced heart failure treated with continuous-flow left ventricular assist device. N. Engl. J. Med..

[4] Goldstein DJ, Meyns B, Xie R, Cowger J, Pettit S, Nakatani T, Netuka I, Shaw S, Yanase M, Kirklin JK (2019). Third annual report from the ISHLT Mechanically Assisted Circulatory Support Registry: a comparison of centrifugal and axial continuous-flow left ventricular assist devices. J. Heart Lung Transplant..

[5] John R, Kamdar F, Liao K, Colvin-Adams M, Boyle A, Joyce L (2008). Improved survival and decreasing incidence of adverse events with the HeartMate II left ventricular assist device as bridge-to-transplant therapy. Ann. Thorac. Surg..

[6] Acharya, D., Loyaga-Rendon, R., Morgan, C. J., Sands, K. A., Pamboukian, S. V., Rajapreyar, I., Holman, W.L., Kirklin, J.K., & Tallaj, J. A. INTERMACS analysis of stroke during support with continuous-flow left ventricular assist devices: risk factors and outcomes. *JACC: Heart Failure* **5**(10), 703–711 (2017). 10.1016/j.jchf.2017.06.014 PMC5743224 28958345

[7] Kislitsina ON, Anderson AS, Rich JD, Vorovich EE, Pham DT, Cox JL, McCarthy PM, Yancy CW (2018). Strokes associated with left ventricular assist devices. J. Card. Surg..

[8] Mehra MR, Goldstein DJ, Uriel N, Cleveland JC, Yuzefpolskaya M, Salerno C, Walsh MN, Milano CA, Patel CB, Ewald GA, Itoh A, Dean D (2018). Two-year outcomes with a magnetically levitated cardiac pump in heart failure. N. Engl. J. Med..

[9] Aaronson KD, Slaughter MS, Miller LW, McGee EC, Cotts WG, Acker MA, Jessup ML, Gregoric ID, Loyalka P, Frazier OH, Jeevanandam V, Anderson AS, Kormos RL (2012). Use of an intrapericardial, continuous-flow, centrifugal pump in patients awaiting heart transplantation. Circulation.

[10] Rogers JG, Pagani FD, Tatooles AJ, Bhat G, Slaughter MS, Birks EJ, Boyce SW, Najjar SS, Jeevanandam V, Anderson AS, Gregoric ID, Mallidi H (2017). Intrapericardial left ventricular assist device for advanced heart failure. N. Engl. J. Med..

[11] Tsukui H, Abla A, Teuteberg JJ, McNamara DM, Mathier MA, Cadaret LM, Kormos RL (2007). Cerebrovascular accidents in patients with a ventricular assist device. J. Thorac. Cardiovasc. Surg..

[12] Morgan JA, Brewer RJ, Nemeh HW, Gerlach B, Lanfear DE, Williams CT, Paone G (2014). Stroke while on long-term left ventricular assist device support: Incidence, outcome, and predictors. ASAIO J..

[13] Harvey L, Holley C, Roy SS, Eckman P, Cogswell R, Liao K, John R (2015). Stroke after left ventricular assist device implantation: Outcomes in the continuous-flow era. Ann. Thorac. Surg..

[14] Parikh NS, Cool J, Karas MG, Boehme AK, Kamel H (2016). Stroke risk and mortality in patients with ventricular assist devices. Stroke.

[15] Willey JZ, Gavalas MV, Trinh PN, Yuzefpolskaya M, Garan AR, Levin AP, Takeda K, Takayama H, Fried J, Naka Y, Topkara VK, Colombo PC (2016). Outcomes after stroke complicating left ventricular assist device. J. Heart Lung Transplant..

[16] Chiang YP, Cox D, Schroder JN, Daneshmand MA, Blue LJ, Patel CB, Devore AD, Bishawi M, Milano CA (2020). Stroke risk following implantation of current generation centrifugal flow left ventricular assist devices. J. Card. Surg..

[17] Adams Jr, H. P., Bendixen, B. H., Kappelle, L. J., Biller, J., Love, B. B., Gordon, D. L., & Marsh 3rd, E. E. Classification of subtype of acute ischemic stroke. Definitions for use in a multicenter clinical trial. TOAST. Trial of Org 10172 in Acute Stroke Treatment. *Stroke*, *24*(1), 35–41 (1993). 10.1161/01.str.24.1.35 7678184

[18] Hannan MM, Husain S, Mattner F, Danziger-Isakov L, Drew RJ, Corey GR, Mahon NG (2011). Working formulation for the standardization of definitions of infections in patients using ventricular assist devices. J. Heart Lung Transplant..

[19] Milano, C. A., Rogers, J. G., Tatooles, A. J., Bhat, G., Slaughter, M. S., Birks, E. J., Mokadam, N.N., Mahr, C., Miller, J.S., Markham, D.W., Jeevanandam, V., Uriel, N., Aaronson, K.D., Vassiliades, T.A., & Pagani, F.D. HVAD: the ENDURANCE supplemental trial. *JACC: Heart Failure*, *6*(9), 792–802 (2018). 10.1016/j.jchf.2018.05.012 30007559

[20] Von Drygalski A, Curtis BR, Bougie DW, McFarland JG, Ahl S, Limbu I, Baker KR, Aster RH (2007). Vancomycin-induced immune thrombocytopenia. N. Engl. J. Med..

[21] Altemeier WA, Fullen WD, McDonough JJ (1972). Sepsis and gastrointestinal bleeding. Ann. Surg..

[22] Izzy S, Rubin DB, Ahmed FS, Akbik F, Renault S, Sylvester KW, Valtkevicius H, Smallwood JA, Givertz MM, Feske SK (2018). Cerebrovascular accidents during mechanical circulatory support: New predictors of ischemic and hemorrhagic strokes and outcome. Stroke.

